# Accuracy of intravitreal injection volume for aflibercept pre-filled syringe and BD Luer-Lok one-milliliter syringe

**DOI:** 10.1186/s40942-022-00375-3

**Published:** 2022-04-05

**Authors:** John-Michael Guest, Brett Malbin, Gary Abrams, Anthony Parendo, Shibandri Das, Chinwenwa Okeagu, Bing X. Ross, Ashok Kumar, Xihui Lin

**Affiliations:** Kresge Eye Institute, Department of Ophthalmology, Visual and Anatomical Sciences, Wayne State University School of Medicine, 4717 St. Antoine Street, Detroit, MI 48201 USA

**Keywords:** Aflibercept, Intravitreal injection, Pre-filled syringe

## Abstract

**Background:**

To evaluate the accuracy of intravitreal injection volume of the pre-filled syringe (PFS) in which aflibercept is packaged compared to the BD Luer-Lok 1-mL syringe.

**Methods:**

Ophthalmologists injected their typical intravitreal volume for aflibercept using either the PFS or BD Luer-Lok 1-mL syringe for 5 times each. The injected fluid was weighed using a micro-scale and converted to volume. The volume of fluid injected was also evaluated when the 0.05 mL line on the PFS was lined up to the tip or base of the dome-shaped plunger.

**Results:**

Injection volume was measured for 12 physicians. The average injected fluid volume was 74.22 ± 15.87 µL for PFS and 53.42 ± 4.61 µL for the BD Luer-Lok 1-mL syringe (*p* < 0.0001). The average deviation in volume injected for the PFS was higher compared to the BD Luer-Lok 1-mL syringe (11.36 µL vs. 3.35 µL, *p* < 0.0001). When the PFS was lined up with the tip of the dome-shaped plunger at the 0.05-mL line, the average injected volume was 71.03% higher.

**Conclusions:**

The intravitreal injection volume and variability using the new PFS were significantly higher than the volume injected using the BD Luer-Lok 1-mL syringe previously used, potentially leading to higher rates of visually significant elevation of intraocular pressures.

## Introduction

Anti-vascular endothelial growth factor (Anti-VEGF) intravitreal injections have revolutionized the treatment of retinal diseases. Intravitreal injections have become an integral part of the treatment of diabetic retinopathy, retinal vein occlusions, and exudative age-related macular degeneration, et al. [1–4]. Aflibercept (Eylea, Regeneron Pharmaceuticals, Tarrytown, NY, USA) is one of the most commonly used intravitreal medications in ophthalmology. Examining Medicare Part B beneficiaries in 2015 showed 866,749 injections of aflibercept administered [5]. Aflibercept accounted for $1.8 billion in Medicare Part B spending [5]. These numbers continue to increase as the amount of intravitreal injections has steadily increased by 6 to 8% per year [6].

The original packaging of the medication consisted of approximately 0.278 mL of aflibercept in a 1-mL vial in which the clinician drew up the medicine and adjusted the plunger to 0.05 mL in a BD Luer-Lok 1-mL syringe. Recently, the medication became available in pre-filled syringes (PFS). The PFS contains 0.09 mL of aflibercept. Subhi el al. described the potential benefits of a lower risk of contamination and shorter medication preparation time with PFS [7]. However, since switching to the PFS, retina physicians at our institution have reported higher frequency of transient central retinal artery occlusion (CRAO) following intravitreal injections with aflibercept. Gallagher et al. also reported a series of five eyes in four patients who developed transient CRAO following intravitreal injections with aflibercept using PFS [8]. All patients had previously received intravitreal injections of the same medication without complications until the change to PFS [8].

We hypothesized that the higher incidence of complications was due to the larger volume being injected with the PFS compared to the BD Luer-Lok 1-mL syringe. The purpose of this study is to measure the actual volume expelled by ophthalmologists who routinely treat patients with intravitreal aflibercept in a simulated injection setting from the PFS and to compare it to the BD Luer-Lok 1-mL syringe when the intended injection volume is 0.05 mL. We found that intravitreal injections with PFS led to larger injection volumes and volume variability compared to those with BD Luer-Lok 1-mL syringe.

## Materials and methods

This experimental study was performed at the Kresge Eye Institute, Detroit, Michigan. The Institutional Review Board at Wayne State University approved the study protocol. Informed consent was not needed as there were no human subjects in our study. Participants were practicing ophthalmologists who performed at least 10 intravitreal injections of aflibercept per month. The ophthalmologists were educated about the proper plunger position while using the PFS (aligning the base of the dome with the target line according to the package insert) immediately prior to conducting this experiment.

### Syringe preparation and measurement

PFS in which aflibercept is packaged from 5 different lot numbers were collected after clinical use and the needles were discarded. Any remaining aflibercept was rinsed out by flushing the syringe with sterile water. The injecting physician drew up approximately 0.15 mL of sterile water with the PFS and a 30-gauge needle was attached. The physician then expressed the extra volume so that the plunger was aligned at his or her typical aflibercept injection location. The remaining volume was then expressed into a 1-mL Eppendorf tube. The weight of each tube was measured with a micro-scale (Sartorius Practum Precision Balance, LABRepCo, Horsham, PA, USA) prior to and after the injection of the sterile water. The weight was converted to volume based on the molecular weight of water at room temperature. The same procedure was performed with the BD Luer-Lok 1-mL syringe (Becton, Dickinson and Company, Franklin Lakes, New Jersey, USA). Each physician performed a total of 10 injections with 5 injections utilizing each syringe type.

### Statistical analysis

The injection volume with the PFS and the BD Luer-Lok 1-mL syringe was expressed in Mean ± S.D. Deviation for each injection was defined as the absolute value of the difference between the injected volume and the mean injection volume for the syringe type. Average deviation between the two syringe types was also compared. Data were analyzed using unpaired Student’s t-test. Statistical significance was set at *p* < 0.05.

## Results

**Fig. 1:**
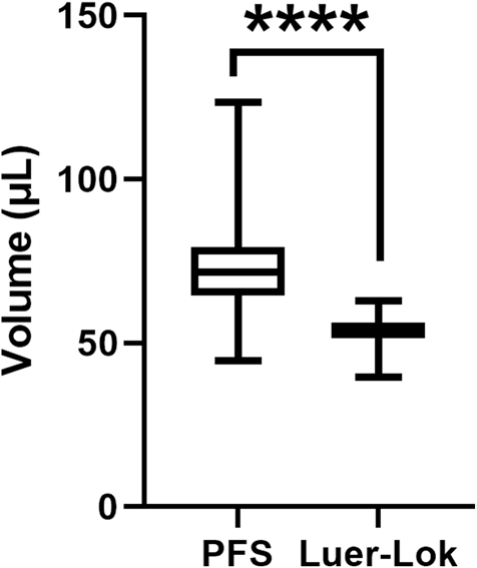
Physician injected volumes with the pre-filled syringe (PFS) in which aflibercept is packaged compared to the BD Luer-Lok 1-mL syringe. Mean ± S.D. ****p < 0.0001 (unpaired Student’s t-test)

Twelve physicians who routinely perform more than 10 intravitreal injections of aflibercept per month performed a total of 120 injections (60 injections using each syringe). The mean ± S.D. injection volume for the BD Luer-Lok 1-mL syringe was 53.42 ± 4.61 µL compared to 74.22 ± 15.87 µL for the PFS (*p* < 0.0001). On average, physicians injected a 38.94% higher volume (20.80 µL) of fluid using the PFS compared to the BD Luer-Lok 1-mL syringe. The average volume deviation was significantly higher by 8.01 µL using the PFS compared to the BD Luer-Lok 1-mL syringe (11.36 µL vs. 3.35 µL, *p* < 0.0001). Figure 1 compares the volume injected and variability between the two types of syringes.

**Fig. 2:**
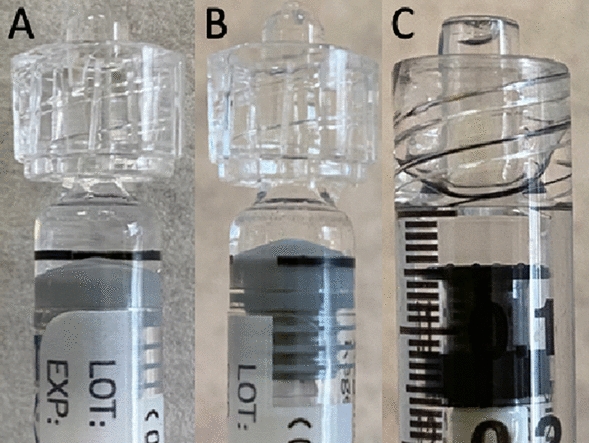
** A** PFS with plunger at tip. **B** PFS with plunger at base. **C** BD Luer-Lok syringe at plunger

**Fig. 3:**
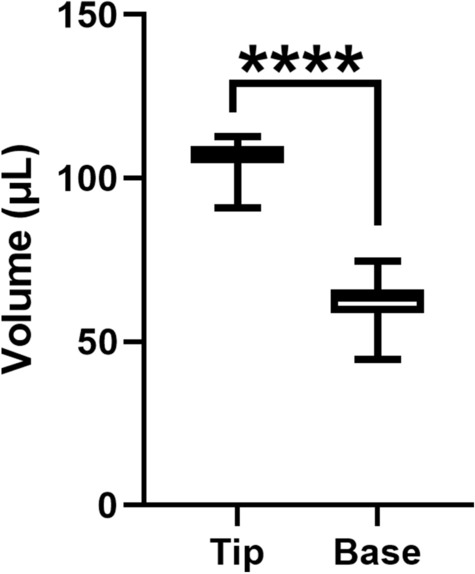
Injection volumes when the plunger is aligned to the tip compared to the base of the dome. Mean ± S.D. ****p < 0.0001 (unpaired Student’s t-test)

In addition to the aforementioned injections, 80 more injections were performed: 40 injections were performed with the plunger aligned at the apex of the dome-shaped plunger (tip) and 40 injections were performed with the plunger aligned at the base of the dome-shaped plunger (base). Figure 2 shows the alignment of the plunger relative to the injection line for the PFS and the BD Luer-Lok 1-mL syringe. Figure 3 shows the comparison of the injection volume when the plunger was aligned with the apex and with the base of the dome. There was a 71.03% higher injection volume when the plunger was aligned with the apex compared to the base of the dome (106.01 µL vs. 61.98 µL, *p* < 0.0001).

## Discussion

Intravitreal injection of medications to treat vitreoretinal diseases have become common practice since the introduction of anti-VEGF therapy to treat eye diseases. The traditional complications of concern were endophthalmitis, lens damage, hemorrhage, and retinal detachment, etc. However, since the aflibercept became available in PFS, severe acute elevation of intraocular pressure leading to vision loss has occurred at an increased rate. In this study, we investigated the injection volume and volume variability using the PFS and found that injections with PFS led to larger injection volumes and volume variability compared to the BD Luer-Lok 1-mL syringe, potentially leading to higher rates of clinically significant elevation of intraocular pressures.

A typical clinical scenario of transient vision loss associated with intravitreal injection occurs immediately after the injection in which the patient experiences a sudden deterioration of visual acuity to no light perception. The intraocular pressure is usually above 45 mmHg. Fundus exam reveals a pale non-perfused optic nerve consistent with CRAO. The vision usually improves to hand motion or better spontaneously within one to two minutes or immediately after anterior chamber paracentesis. Over the next 5 to 10 min, the patient usually regains baseline vision.

Historical context exists for the injection of larger intravitreal volumes. Arikan et al. reported that pressure spikes following intravitreal injections were more common and severe following injection of 0.10 mL versus 0.05 mL of triamcinolone [9]. Even with large intravitreal volume injections, transient CRAO remains rare [10]. Acute vision loss is exceedingly rare following intravitreal injection volume of 0.05 mL, but occasionally occurs in patients with significant baseline optic nerve pathology such as advanced glaucoma or those with high baseline intraocular pressure [11, 12]. Higashide et al. described a 2% rate of CRAO in patients receiving 0.05 mL intravitreal anti-VEGF injections for neovascular glaucoma [13]. Good et al. reported that following intravitreal anti-VEGF injection, patients with glaucoma have a higher incidence of elevated intraocular pressure and the pressure elevation sustained for longer period of time than patients without glaucoma [14]. The anatomy of the eye may also play a role in increased risk of complications. Some studies have shown that eyes with a shorter axial length are at increased risk of intraocular pressure spikes [15, 16].

When compared to the small-volume syringe used in the previous iteration of aflibercept package, the large increase and variability in injection volume with PFS are significant findings [17]. Since aflibercept was packaged in PFS, a higher rate of transient vision loss was reported. Gallagher et al. described 5 cases of CRAO following intravitreal aflibercept injections after switching to the new PFS [8]. In a letter to the editor, Gallagher described a larger injection volume with the PFS compared to the previously used BD Luer-Lok 1-mL syringe [8]. Our data suggest the injection volumes using the BD Luer-Lok 1-mL syringes are relatively accurate and consistent, making them a good control arm for comparison with the new PFS. In an eye with a healthy optic nerve and normal baseline intraocular pressure, a higher intravitreal injection volume would probably be well tolerated. However, it is likely that a higher proportion of patients will experience a visually significant intraocular pressure rise with the additional 38.94% volume injected with the PFS.

Several aspects of the new syringe design are likely responsible for the higher injection volumes and variability. Firstly, the plunger in the PFS is dome-shaped while that in the BD Luer-Lok 1-mL syringe is flat (Fig. 2). Secondly, the diameter of the PFS is larger compared to that of the BD Luer-Lok 1-mL syringe. Therefore, small differences in plunger alignment will yield large volume variations. Finally, the PFS lacks a clearly defined alignment point at the base of the dome. In our evaluation of the plunger position, even if it was meticulously aligned at the base of the dome, there was still an average intravitreal injection volume of 61.98 µL—over 20% greater than the anticipated volume of 50 µL. If one incorrectly aligned the apex rather than base of the dome with the target line, 71.03% more volume would be injected. Although the package insert clearly instructs aligning the base of the dome with the target line, it is possible some clinicians might align closer to the apex to error on the side of caution to ensure their patients receive at least the full intravitreal dose. Given the large volume difference between the alignment at the base and the apex of the dome, a small change in plunger position can lead to a drastic change in injection volume and intraocular pressure. Gallagher et al. noted a similar increased variability of PFS compared to the BD Luer-Lok 1-mL syringe whereby small changes in the position of the plunger cause greater changes in volume injected in the PFS [8]. Goldberg et al. also reported in the American Academy of Ophthalmology 2021 annual meeting that the dosing variability of the PFS is likely due to the large diameter of the syringes and the wide thickness of dose marks [18]. Loewenstein et al. previously evaluated pre-filled ranibizumab (Lucentis, Genentech, South San Francisco, CA, USA ) syringes and found they are more accurate than the 1-mL syringes [19]. These findings are attributed to the flat shape of the plunger and the clearly demarcated syringe volume line. In addition, the diameter of the PFS in which ranibizumab is packaged is smaller. Hinkle et al. compared the stopper position and injection volume in dome shaped PFS in which ranibizumab or aflibercept is packaged and found the larger variation in volume of the PFS in which aflibercept is packaged is due to the larger diameter of the PFS [20]. These findings suggest plunger construction and the location of the target line in the PFS in which aflibercept is packaged are likely the cause of the inconsistency in injection volumes.

The main limitation of the study is the *in vitro* nature of the injections. It is possible that true injected volumes in the eye are different given there is frequently outflow through the sclerotomy site created by the needle. This may modulate some of the pressure increase from the increased volumes. However, the higher expressed volume with the PFS is likely true *in vivo*. One can argue that some variability is unavoidable when the volume injected is 0.05 mL given the precision required to measure exactly the same volume each time. However, the BD Luer-Lok 1-mL syringe provides good accuracy and low variability over repeated injections by different examiners. This syringe can serve as a good control syringe for the expected volume injected and variability. It appears, on average, clinicians were able to inject within 5 µL of the target volume using the BD Luer-Lok 1-mL syringe. In comparison, the variability when using the PFS is significantly higher.

## Conclusions

Physicians who regularly perform intravitreal injections, on average, injected a significantly higher volume with higher variability using the PFS compared to the BD Luer-Lok 1-mL syringe. We recommend all physicians using PFS re-evaluate their injection approach and make necessary adjustments to avoid visually significant intraocular pressure elevations after injections. The design of the PFS in which aflibercept is packaged should also be reconsidered.

## Data Availability

The datasets used and/or analyzed during the current study are available from the corresponding author on reasonable request.

## Abbreviations

PFS: Pre-filled syringe
Anti-VEGF: Anti-vascular endothelial growth factor
CRAO: Central retinal artery occlusion
